# Viromes of 15 Pepper (*Capsicum annuum* L.) Cultivars

**DOI:** 10.3390/ijms231810507

**Published:** 2022-09-10

**Authors:** Yeonhwa Jo, Hoseong Choi, Jeong Hun Lee, Sang Hyun Moh, Won Kyong Cho

**Affiliations:** 1 College of Biotechnology and Bioengineering, Sungkyunkwan University, Suwon 16419, Korea; 2 Plant Genomics and Breeding Institute, Seoul National University, Seoul 08826, Korea; 3 Plant Cell Research Institute of BIO-FD&C Co., Ltd., Incheon 21990, Korea

**Keywords:** *Capsicum annuum*, pepper, RNA sequencing, virome, virus

## Abstract

Pepper (*Capsicum annuum* L.) plants produce berry fruits that are used as spices. Here, we examined the viromes of 15 pepper cultivars through RNA sequencing. We obtained 1,325 virus-associated contigs derived from 8 virus species. Bean broad wilt virus 2 (BBWV2) and cucumber mosaic virus (CMV) were identified as the major viruses infecting pepper plants, followed by potato virus Y, bell pepper endornavirus, and hot pepper endornavirus. The proportion of viral reads in each transcriptome ranged from 0.04% to 24.5%. BBWV2 was the dominant virus in seven cultivars, whereas CMV was dominant in five cultivars. All the bell pepper cultivars showed severe viral disease symptoms, whereas the commercially developed hot pepper cultivars were asymptomatic or had mild symptoms. In addition, 111 complete viral segments were obtained from 7 viruses. Based on the obtained viral genomes, the genetic relationship between the identified viruses and quasispecies of BBWV2 and CMV in each pepper plant was determined. Newly designed primers for nine viruses confirmed the results of RNA sequencing. Taken together, this study, for the first time, provides a comprehensive overview of viromes in 15 major pepper cultivars through RNA sequencing.

## 1. Introduction

The pepper plant *Capsicum annuum* L., a member of the nightshade family Solanaceae, is known to produce berry fruits [1]. Peppers are cultivated for their pungency and used as spices in many cuisines across the world [2]. The plant originated in Bolivia and was first cultivated in Mexico [3]. Many pepper cultivars spread worldwide during the Columbian Exchange; Portuguese traders introduced the plant to Asian countries. To date, five pepper species have been domesticated. The most commonly cultivated pepper groups are bell peppers, sweet peppers, and hot peppers.

In Korea, bell peppers and hot peppers are widely cultivated. Hot peppers are consumed as fresh fruits or are dried to produce pepper powder. Those hot peppers can be divided into red hot pepper and sweet pepper based on their spiciness. While most other countries use the term “paprika” for chili or hot peppers, in Korea, “paprika” is used interchangeably with bell pepper to refer to the large sweet peppers in a variety of colors such as green, orange, yellow, and red. The production of bell peppers, mostly consumed as fresh fruit, has been increasing in Korea. According to the Korea Seed and Variety Service registry, approximately 600 hot pepper and 60 bell pepper cultivars have been developed to date by more than 50 seedling companies. Most pepper cultivars grown in Korea are of the red hot pepper variety, used to produce red hot pepper powders, one of the most important spices in Korean cuisine, used in kimchi, pastes, and other side dishes [4].

Pepper plants are usually cultivated from April to October in Korea, and red hot pepper fruits are harvested from July to October. Diseases, such as phytophthora blight and anthracnose, and harmful insects, such as whitefly, oriental tobacco budworm, and thrip, cause serious damage to the quality and quantity of pepper production. Therefore, a variety of pesticides are regularly sprayed on pepper plants. However, excessive pesticide use poses a major risk to human and environmental health.

In addition to harmful bacteria, fungi, and insects, several viruses are also known to infect pepper plants. For example, DNA viruses belonging to the genus *Geminivirus* and RNA viruses with single- or double-stranded RNA genomes are major viruses that infect pepper plants [5]. Viroids such as columnea latent viroid and pepper chat fruit viroid also infect these plants [6].

In Korea, the most frequently identified viruses infecting pepper plants are the cucumber mosaic virus (CMV), broad bean wilt virus 2 (BBWV2), and tomato spotted wilt virus (TSWV) [7]. Hot pepper endornavirus (HPEV), pepper cryptic virus 1 (PCV1), and pepper cryptic virus 2 (PCV2) have also recently been identified [8]. However, unlike in other Asian countries, no DNA viruses or viroids infecting pepper plants have yet been identified in Korea.

While some farmers grow pepper seedlings, most purchase them from different nurseries. In many cases, nurseries cultivate multiple plant varieties within a limited space, such as in a greenhouse, potentially increasing the frequency of viral infections. In this study, we examined the viromes of 15 different pepper cultivars derived from 5 major nurseries in Korea, via RNA sequencing, followed by intensive bioinformatic analyses.

## 2. Results

### 2.1. Collection of Leaf Samples from 15 Different Pepper Cultivars

We collected 15 pepper cultivars from 5 nurseries in Korea (Table 1). The nurseries were located in Anyang (four cultivars), Hampyeong (five cultivars), Ilsan (four cultivars), Pusan (one cultivar), and Uljin (one cultivar). Of these, six were highly pungent red hot peppers, five were sweet peppers with a low level of pungency, and four were bell peppers.

### 2.2. Viral Disease Symptoms in Different Pepper Cultivars

**Figure 1:**
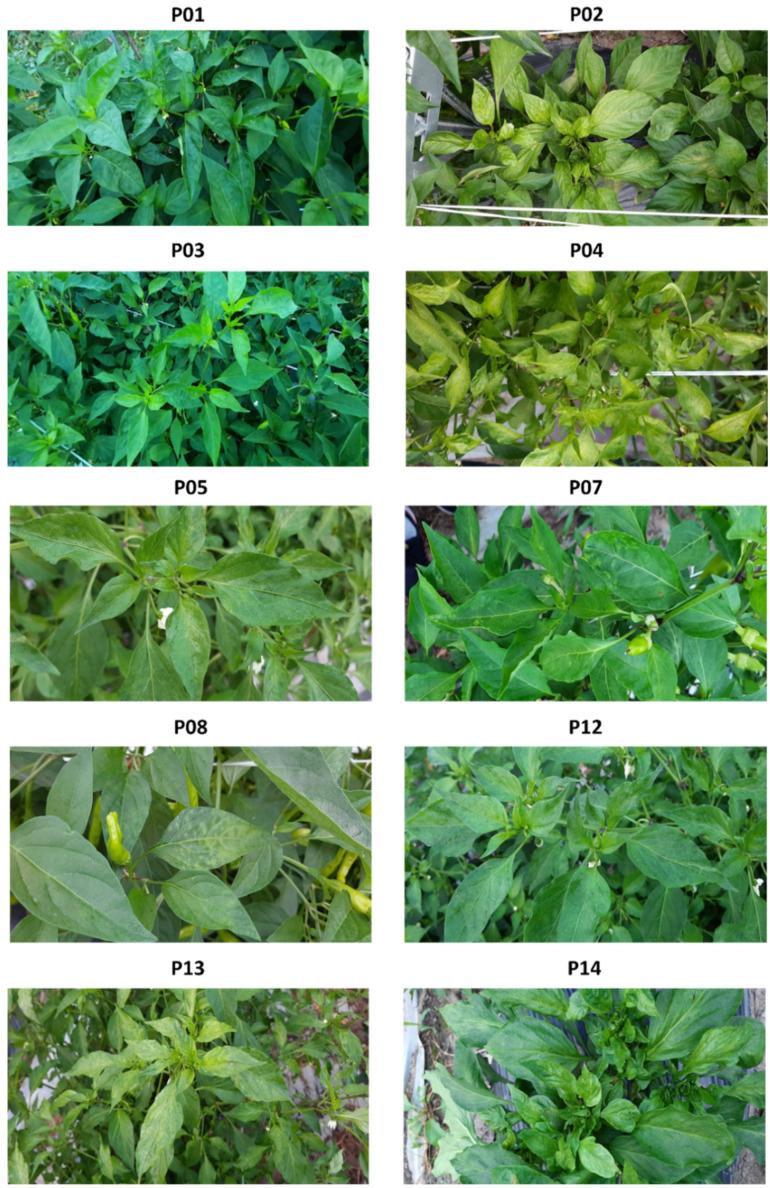
Images of 10 representative pepper cultivars. Few cultivars, such as P01 and P03, did not show any visible viral disease symptoms, while all paprika plants, such as P02 and P14, displayed very severe disease symptoms, such as stunting, and abnormal growth. Most pepper cultivars displayed mild symptoms, such as chlorosis, curling, mosaic, mottling, and vein banding.

All the bell pepper plants showed severe viral disease symptoms such as stunting, mosaic pattern, and curling (Figure 1). These plants did not grow well and produced a small number of fruits. Among the hot pepper cultivars, P13 (Cheongyang) exhibited the strongest disease symptoms, including a mosaic pattern, mottling, chlorosis, and leaf distortion. However, the Cheongyang cultivar produced a high number of normal-quality pepper fruits. The remaining pepper cultivars showed mild viral disease symptoms or were asymptomatic (P01 and P03).

### 2.3. Identification of Virus-Associated Contigs from 15 Different Pepper Cultivars

**Figure 2:**
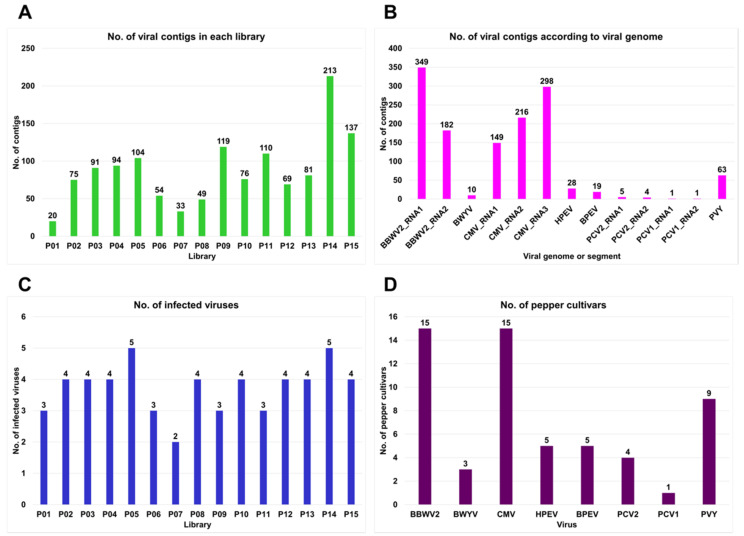
Number of identified virus-associated contigs and viruses from 15 different pepper cultivars: (**A**) number of viral contigs identified from each library; (**B**) number of viral contigs according to identified viral genome or segment; (**C**) number of viruses identified from each library; (**D**) number of pepper cultivars in which individual viruses were identified.

Regardless of the viral disease symptoms, we randomly selected individuals from different pepper cultivars. From each individual, we harvested five different leaf samples and extracted total RNA. We deleted ribosomes from these total RNAs and generated 15 different libraries representing individual pepper cultivars. By sequencing RNA followed by *de novo* transcriptome assembly using the Trinity program, we obtained 15 pepper transcriptomes. Each transcriptome was subjected to a BLASTX against the viral protein database. The identified virus-associated contigs were further subjected to a BLASTX against non-redundant protein databases to filter out non-viral sequences. Finally, 1325 virus-associated contigs were obtained. P14 contained the highest (213), and P01 showed the lowest (20) number of virus-associated contigs (Figure 2A).

The identified virus-associated contigs were derived from eight RNA viruses, four of which (BBWV2, CMV, PCV2, and PCV1) were identified in this study and known to have multipartite genomes. For example, BBWV2, PCV2, and PCV1 had two RNA segments, whereas CMV had three. In total, 663 virus-associated contigs were derived from three CMV RNA segments: RNA1 (149 contigs), RNA2 (216 contigs), and RNA3 (298 contigs) (Figure 2B). A total of 531 virus-associated contigs were derived from two BBWV2 segments: RNA1 (349 contigs) and RNA2 (182 contigs). The other four RNA viruses, Beet western yellows virus (BWYV) (10 contigs), HPEV (28 contigs), bell pepper endornavirus (BPEV) (19 contigs), and potato virus Y (PVY) (63 contigs), were observed to have a monopartite genome.

All the examined pepper cultivars were coinfected with at least two viruses (Figure 2C). The number of infected viruses in each cultivar ranged from two (P07) to five (P05 and P14). The cultivars P01, P06, P09, and P11 were infected with three different viruses, whereas the remaining eight cultivars were infected with four different viruses.

We also examined the number of pepper cultivars infected with the individual virus (Figure 2D). BBWV2 and CMV were detected in 15 and 13 pepper cultivars, respectively. PVY infected nine cultivars, whereas BPEV and HPEV infected five cultivars. BWYV was detected in three cultivars, whereas only one cultivar (P14) was infected with PCV1.

**Figure 3:**
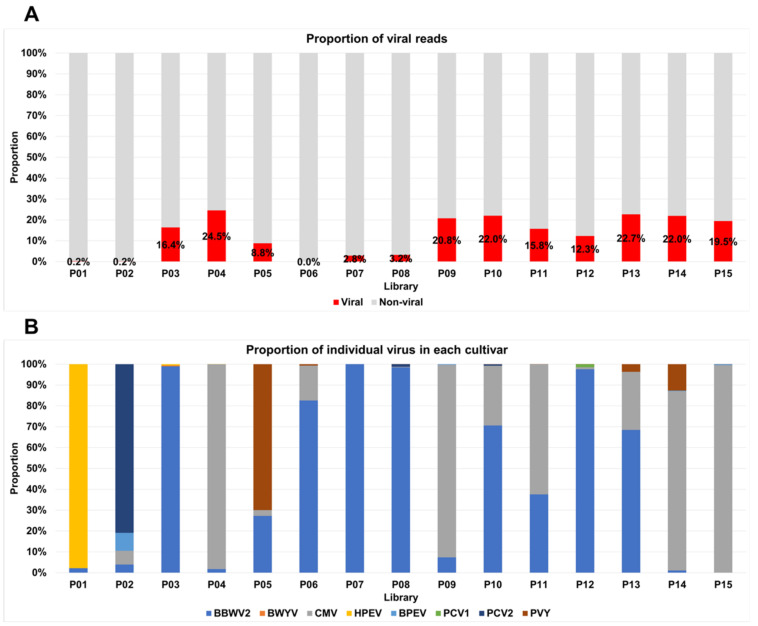
The proportion of viral reads in each library and the proportion of identified viruses in each library: (**A**) the proportion of viral reads (red color) as compared to non-viral reads (gray color); (**B**) the proportion of identified viruses in each library. Only viral reads assigned to the identified viruses were used for the calculation of virus proportion in each library.

The proportion of viral reads in each transcriptome ranged from 0.04% (P06) to 24.5% (P04) (Figure 3A). The proportion of viral reads for three cultivars (P01, P02, and P06) was less than 1%, whereas six cultivars (P04, P09, P10, P13, P14, and P15) had a viral read proportion of more than 20%. HPEV and PVY were predominant in P01 and P05, respectively, whereas PCV2 was the dominant virus in P02 (Figure 3B). BBWV2 was the dominant virus in seven cultivars (P03, P06, P07, P08, P10, P12, and P13), and CMV exhibited its dominance in five cultivars (P04, P09, P11, P14, and P15).

### 2.4. Viral Genome Assembly

**Figure 4:**
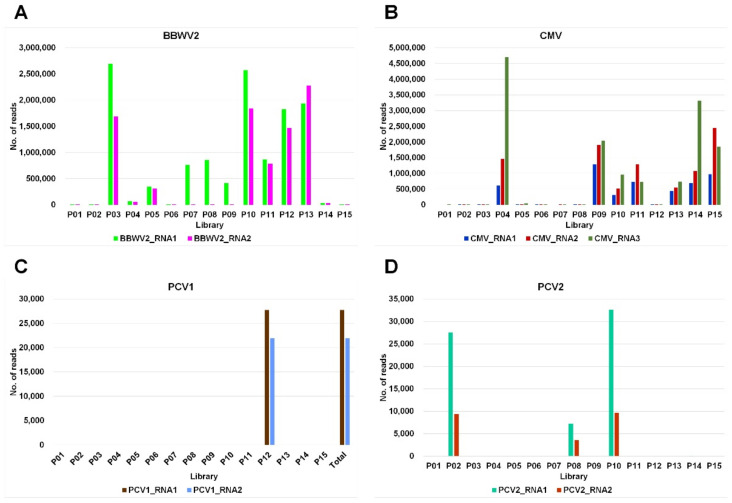
The number of viral reads in each library derived from four viruses composed of multiple viral segments. The number of viral reads in each library derived from BBWV2 composed of two RNA segments (**A**), CMV composed of three RNA segments (**B**), PCV1 composed of two RNA segments (**C**), and PCV2 composed of two RNA segments (**D**).

**Figure 5:**
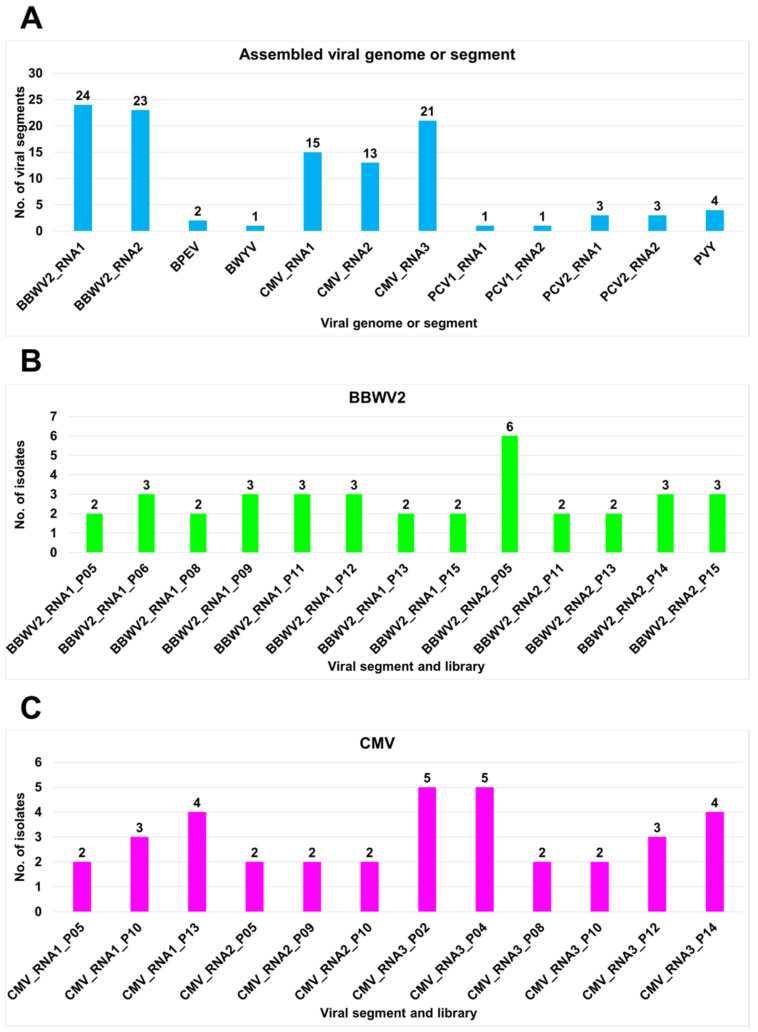
Number of assembled viral genomes: (**A**) total number of viral genomes or viral segments *de novo* assembled from RNA sequencing; (**B**) number of assembled viral variants for BBWV2 in each library; (**C**) number of assembled viral variants for CMV in each library. For viruses with multiple RNA segments, the number of assembled viral genomes is indicated by each viral segment.

We obtained 111 complete viral segments for 7 viruses by *de novo* transcriptome assembly from 15 samples (Table S1). When the assembled viral genome covered all open reading frames (ORFs), we regarded it as the complete viral genome. Out of the eight identified viruses, four had multipartite genomes. Therefore, we examined the number of assembled segments for each virus (Figure 4). We obtained the highest number of BBWV2 RNA1 segments (24) followed by BBWV2 RNA2 (23) (Figure 4A). For CMV, the highest number of segments were obtained from CMV RNA3 (21), followed by CMV RNA1 (15) and CMV RNA2 (13) (Figure 4B). We obtained the same number of segments for each virus for PCV1 (1) and PCV2 (3; Figure 4C,D). We obtained the complete genomes for BPEV (2), BWYV (1), and PVY (4; Figure 5A). However, we did not obtain a complete genome sequence for HPEV.

For BBWV2 and CMV, the complete genome sequences of several variants (isolates) were identified from the same pepper sample (Figure 5B,C). In each pepper sample, the number of BBWV2 RNA1 variants ranged from two to three, while the number of BBWV2 RNA2 variants ranged from six to three (Figure 5B). The numbers of variants of BBWV2 RNA1 and BBWV2 RNA2 were not consistent among the samples. For example, in P05, there were two variants of BBWV2 RNA1 and five variants of BBWV2 RNA2. We identified 20 variants of BBWV2 RNA1 and 16 variants of BBWV2 RNA2 from eight samples.

Similarly, we identified 36 CMV genome sequences, CMV RNA1 (9 sequences), CMV RNA2 (6 sequences), and CMV RNA3 (21 sequences) (Figure 5C). Interestingly, the number of variants for each CMV RNA segment varied among the pepper samples. The number of CMV RNA1 variants ranged from two to four, while the number of samples for CMV RNA2 variants ranged from two to three samples. For CMV RNA3, the number of variants in each sample ranged from two to five. However, the number of variants among the CMV RNA segments in the same sample was not consistent.

**Figure 6:**
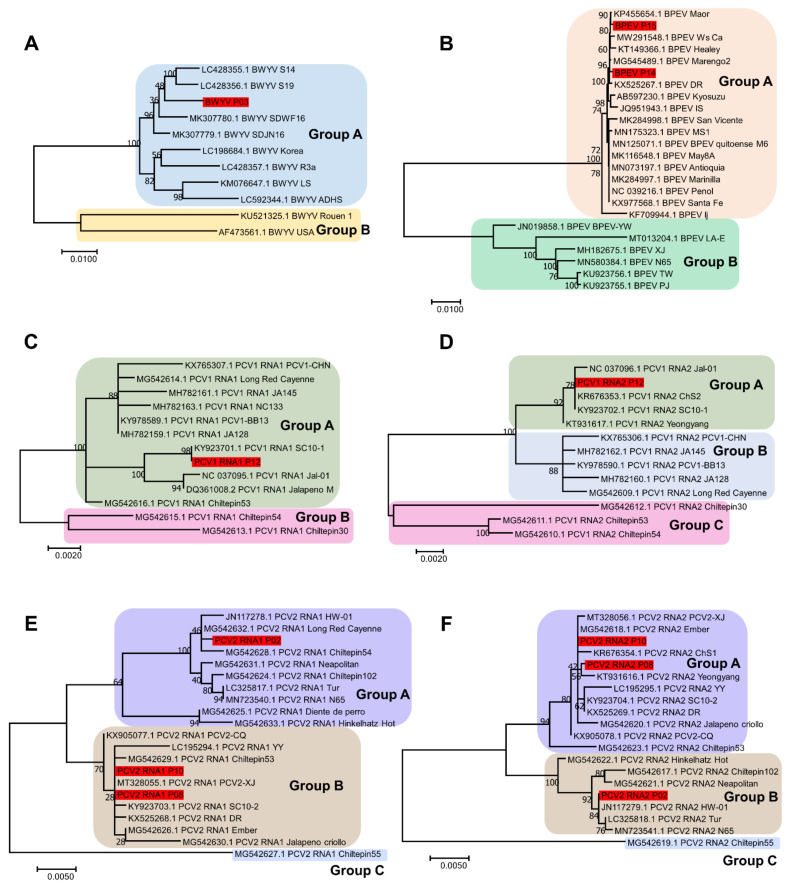
Phylogenetic tree of four viruses infecting pepper cultivars; BWYV, BPEV, PCV1, and PCV2: (**A**) phylogenetic tree of complete genome sequences of BWYV (**A**), BPEV (**B**), PCV1 RNA1 segment (**C**), PCV1 RNA2 segment (**D**), PCV2 RNA1 segment (**E**), and PCV2 RNA2 segment (**F**). Red-colored boxes indicate the assembled viral genome obtained from this study.

Based on the obtained viral genomes, we analyzed the phylogenetic relationships of the identified viral variants with known isolates for each virus. For BWYV, only a single variant, P03, was identified in this study. This variant showed sequence similarity to known BWYV isolates S14 and S19, which were identified in spinach in Japan. The phylogenetic tree using 11 BWYV isolates showed that BWYV variant P03 belonged to group A, possessing nine isolates of BWYV, whereas group B contained two BWYV isolates from France and the United States (Figure 6A).

Although we identified two alphaendornaviruses, HPEV and BPEV, only two complete genomes of the BPEV variants, P14 and P15, were obtained. The phylogenetic tree using 24 BPEV isolates demonstrated that P14 and P15 belonged to group A containing 18 BPEV isolates (Figure 6B). Most of the BPEV isolates in group A were closely related. In contrast, the six BPEV isolates in group B were distantly related.

We identified a single PCV1 variant in the P12 library. The PCV1 genome consists of two RNA segments: RNA1 and RNA2. The phylogenetic tree based on the 13 PCV1 RNA1 sequences revealed two groups of PCV1 isolates (Figure 6C). RNA1 of PCV1 variant P12 showed strong sequence similarity to the known PCV1 isolate SC10-1 identified in Korea, and both the Korean isolates belonged to group A. A phylogenetic tree using 13 sequences of PCV1 RNA2 identified three groups of PCV1 isolates (Figure 6D). Group A included four isolates from Korea (P12, ChS2, SC10-1, and Yeongyang) and one isolate (Jal-01) from the United States. The five isolates in group B were closely related. While the three PCV1 isolates in group C were identified from the same country (Mexico), they were distantly related.

For PCV2, composed of two RNA segments, we obtained three and two complete genome sequences for RNA1 and RNA2, respectively. We generated two phylogenetic trees based on the 21 PCV2 RNA1 and 20 PCV2 RNA2 sequences (Figure 6E,F). Both phylogenetic trees revealed the presence of three groups of PCV2 isolates. Interestingly, the PCV2 isolate Chiltepin55, identified in Mexico, was genetically distant from the other PCV2 isolates. Group A for PCV2 RNA1 contained 10 isolates, including the P02 isolate identified in this study, whereas group B contained 10 isolates, including variants P08 and P10 detected in this study (Figure 6E). For RNA2 of PCV2, group A contained 12 PCV2 isolates, including P08 and P10 found in this study, whereas 7 isolates belonged to group B, including P02 identified in this study (Figure 6F).

**Figure 7:**
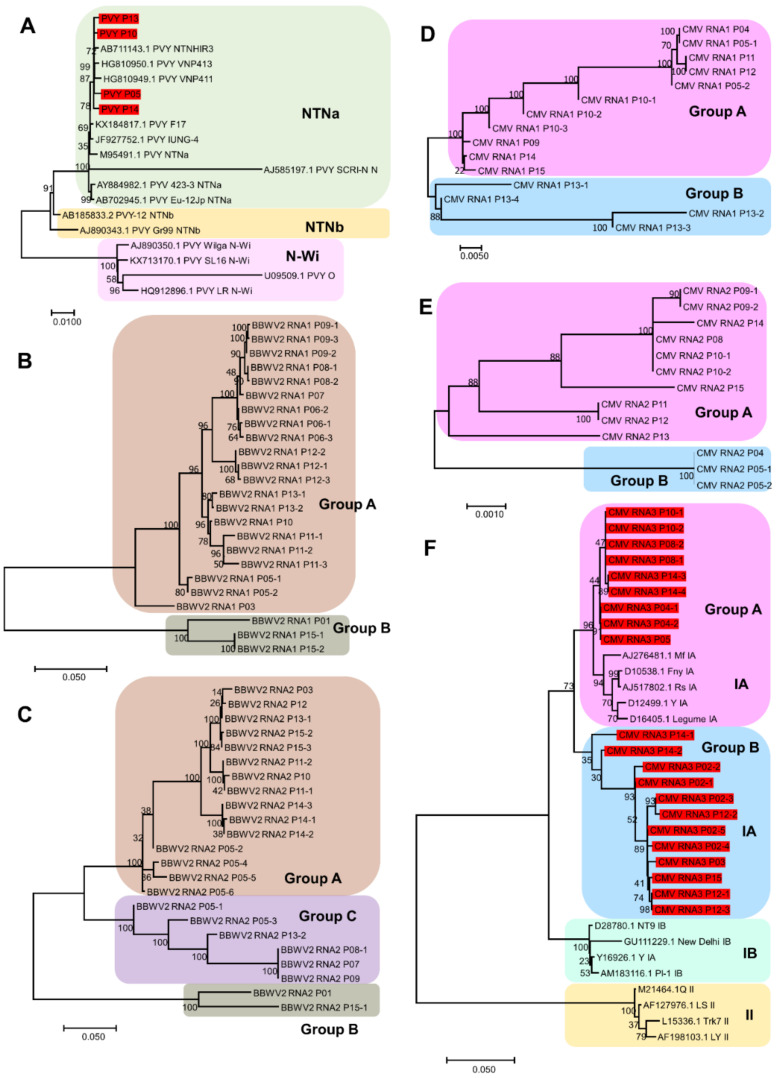
Phylogenetic tree of viruses, PVY, CMV and BBWV2, infecting pepper cultivars: phylogenetic tree of complete genome sequences of PVY (**A**), BBWV2 RNA1 segment (**B**), BBWV2 RNA2 segment (**C**). CMV RNA1 segment (**D**), CMV RNA2 segment (**E**), CMV RNA3 segment (**F**). The viral genome sequences in (**A**,**B**) derived from this study were indicated by red-colored boxes.

The genomes of four PVY variants were obtained in this study. According to a previous study, several strains of PVY have been identified [9]. We included nine known PVY strains for the construction of a phylogenetic tree of PVY based on a previous study [10]. The phylogenetic tree using 19 PVY isolates showed three groups, namely NTNa, NTNb, and N-Wi (Figure 7A). The four PVY variants identified in this study and two isolates from potatoes in Vietnam and Japan were grouped together with the three known PVY isolates belonging to the NTNa strain.

For BBWV2, which is composed of two RNA segments, two different phylogenetic trees were constructed based on the individual RNA segments obtained in this study (Figure 7B,C). The phylogenetic tree of BBWV2 RNA1 using the 24 sequences identified in this study showed two groups of BBWV2 isolates (Figure 7B). Group A contained 21 sequences, whereas group B contained 3 sequences from P01 and P15 samples. In general, variants from the same plant were observed to be closely related to each other. The phylogenetic tree based on the 23 sequences of BBWV2 RNA2 displayed three groups of BBWV2 variants (Figure 7C). P01, P15-1, P15-2, and P15-3 variants belonged to group A. Group A included 15 variants, whereas group B contained 6 variants. The P05 samples yielded six BBWV2 RNA2 sequences. Of these, four belonged to group A, and two belonged to group B.

We obtained 15, 13, and 21 sequences for CMV RNA1, RNA2, and RNA3, respectively. We constructed three phylogenetic trees based on individual RNA segments (Figure 7D–F), which were used to identify two groups of CMV variants. None of these groups contained the same list of CMV variants. In general, variants from the same sample were closely related. However, some variants from the same sample were observed to be distantly related to each other. For example, the four variants of CMV RNA1 from the P13 sample in group B were genetically different. In contrast, P04, P05-1, and P05-2 of CMV RNA2 as well as P04-1, P04-2, and P05 of CMV RNA3 were closely related, although they were derived from two different samples. CMV isolates can be classified into two major subgroups, I and II. Subgroup I can be further divided into IA and IB. We included RNA3 sequences of 13 known CMV isolates representing CMV subgroups for the phylogenetic tree construction based on a previous study [11]. Although 21 CMV RNA sequences in this study were divided into two groups (group A and group B), all the 21 CMV RNA3 variants were grouped together with the 5 CMV isolates belonging to subgroup IA (Figure 7F).

**Figure 8:**
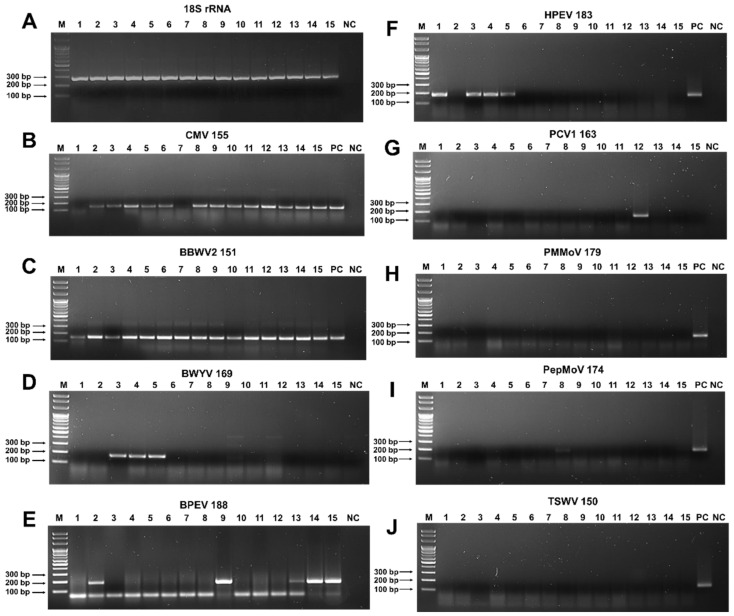
Detection of viruses infecting pepper plants by RT-PCR: (**A**) RT-PCR results using 18S rRNA specific primers to confirm the quality of extracted total RNAs. RT-PCR results using specific primers for CMV (**B**), BBWV2 (**C**), BWYV (**D**), BPEV (**E**), HPEV (**F**), PCV1 (**G**), PMMoV (**H**), PepMoV (**I**), and TSWV (**J**). C indicates positive control. The same total RNAs used for RNA sequencing were used for RT-PCR. The number with the virus name indicates the expected size of the RT-PCR amplicon. PC and NC indicate positive and negative controls, respectively.

To confirm the results of RNA sequencing, we conducted RT-PCR using virus-specific primers. To detect viruses infecting pepper plants, we designed different primer pairs for nine viruses (Table 2). A primer pair amplifying a partial sequence of 18S rRNA for *C. annuum* (Ca18SrRNA) was used as a positive housekeeping gene for RT-PCR [12]. The designed primers can be used in three different methods: RT-PCR, real-time RT-PCR, and recombinase polymerase amplification (RPA). The same total RNAs used for RNA sequencing were used as templates for RT-PCR. Using a primer pair specific to 18S ribosomal RNA for pepper plants, we confirmed the quality of the total RNAs (Figure 8A). Using CMV-specific primers, we detected CMV in all the pepper samples, except P01 and P07 (Figure 8B). BBWV2 infected all the pepper plants, as revealed by RT-PCR (Figure 8C). BWYV was detected in three samples (P03, P04, and P05) (Figure 8D). In addition, we identified two alphaendornaviruses (BPEV and HPEV), which had similar genome organization and high sequence similarity. Therefore, we designed primer pairs that could selectively amplify the virus-specific sequences. Using the BPEV-specific primer pairs, we detected BPEV in the P02, P09, P13, P14, and P15 samples (Figure 8E), whereas HPEV was detected in the P01, P03, P04, and P05 samples (Figure 8F). Using the PCV1-specific primer pairs, we detected only PCV1 in the P12 sample (Figure 8G). In addition, we carried out RT-PCR to detect pepper mild mottle virus (PMMoV), pepper mottle virus (PepMoV), and TSWV, which were not identified with RNA sequencing (Figure 8H–J). The RT-PCR results revealed that none of the pepper samples was infected by PMMoV, PepMoV, or TSWV. These observations were consistent with the RNA sequencing results.

## 3. Discussion

Viruses and viroids that infect pepper plants have been intensively examined in many countries [5,6,8]. However, viruses that infect different pepper cultivars have not yet been investigated. In this study, we, for the first time, revealed comprehensive viromes of 15 pepper cultivars cultivated in Korea, through sequencing.

We found that the most prominent viruses infecting pepper plants in Korea were BBWV2 and CMV. Both RNA sequencing and RT-PCR results revealed that all 15 cultivars were infected by BBWV2, while 13 cultivars were infected by CMV. Infection of BBWV2 has been widely reported in cultivated pepper plants in Korea [13]. However, there exists a limited number of studies reporting the infection of pepper plants by BBWV2 in other countries. For example, a previous study reported BBWV2 infection in pepper plants in the Czech Republic, using a double-antibody sandwich (DAS)–ELISA analysis [14]. We assume that either BBWV2 infection in pepper plants has not been intensively studied, or this virus is not a prominent source of infection in pepper plants in other countries.

In addition to hot pepper cultivars, we examined viruses infecting four bell pepper cultivars: P02, P09, P14, and P15. As expected, BPEV was identified in all the bell pepper cultivars. To our knowledge, this is the first study to report BPEV infection in bell pepper plants in Korea. In addition, we identified BPEV in a single hot pepper (Cheongyang cultivar, P13). Infection of hot peppers with BPEV has not been previously reported. This is the first study to report BPEV infection in hot pepper cultivars. HPEV was first identified in hot peppers in Korea [15] followed by hot peppers in China [16]. Interestingly, in this study, HPEV was identified in two hot pepper cultivars (P01 and P03) and two sweet pepper cultivars (P04 and P05) but not in bell pepper cultivars. Based on this result, we inferred that bell pepper cultivars could be the main hosts for BPEV in Korea, although there was an exception (P13). In addition, hot pepper and sweet pepper cultivars are the main hosts of HPEV. Both BPEV and HPEV are vertically transmitted via seeds [17]. Thus, it is likely that HPEV infecting sweet pepper cultivars could originate from breeding hot pepper cultivars. Both BPEV and HPEV belong to the genus *Alphaendornavirus* in the family *Endornaviridae* [18]. Their genome organization is reported to be quite similar to each other, and the known HPEV isolate has shown low sequence similarity (94% query cover and 72% identity) to the BPEV isolate Yolo Wonder. It was, therefore, difficult to distinguish between them without knowledge of their genome sequences.

According to a previous study in 2005, the most common viruses infecting pepper plants in Korea were CMV (19.8%) followed by PepMoV (13.4%), PMMoV (1.1%), BBWV2 (3.8%), and TMGMV (0.5%) [7]. Some viruses known to infect pepper plants, such as alfalfa mosaic virus (AMV), PVY, tobacco mosaic virus (TMV), and pepper vein chlorosis virus (PVCV), were not identified in our study. BBWV2 was the most prominent virus, followed by CMV; however, PepMoV, PMMoV, and TMGMV were not detected in this study. This is similar to our previous study, which also showed that CMV and BBWV2 were the two major viruses infecting overwintering pepper fruits [8]. Thus, CMV and BBWV2 might be the two major viruses infecting pepper plants in Korea.

PVY was initially identified in bell peppers (paprika) in Korea [19]. In our study, PVY was identified in sweet peppers (P05 and P10), hot peppers (P13), and bell peppers (P14 and P15). These results suggest that PVY could be an important virus infecting pepper plants owing to its presence in diverse pepper cultivars. Phylogenetic analyses revealed that the four PVY variants identified in this study were closely related to the PVY-infecting potato plants derived from Vietnam and Japan. Therefore, PVY variants infecting pepper plants might also be derived from potato plants. Generally, pepper and potato plants are cultivated during similar periods in Korea. Thus, aphid species identified as the main vectors of PVY can easily transmit PVY to the two plant species. In addition, we revealed that all the PVY variants in this study belong to the known PVY strain NTNa, which is one of the common PVY recombinants [9].

Due to differences in pepper samples and collection regions, other known viruses infecting pepper plants, such as PepMoV, PMMoV, and TMGMV, were not detected in this study. Moreover, in contrast to other Asian countries such as India [5] and Vietnam [6], we did not identify any DNA viruses and viroids, suggesting the importance of quarantine in preventing the introduction of these viruses and viroids to pepper plants in Korea.

In contrast to most previous studies that have used RT-PCR and ELISA assays to identify viruses infecting pepper plants in Korea [7], we used RNA sequencing to identify the known and unknown viruses infecting pepper plants. In a single study, we identified eight different viruses that infected pepper plants and provided a detailed viral composition for each pepper cultivar. Moreover, RNA sequencing enabled us to obtain 111 complete viral genome sequences.

It is noteworthy that the small number of reads derived from RNA sequencing might have been caused by cross-contamination from another library. For example, three reads for CMV RNA3 in the P01 cultivar and one read each for CMV RNA2 and CMV RNA3 could be cross-contaminants. The RT-PCR results demonstrated that CMV was not present in the P01 and P07 cultivars.

Due to the coinfection of different viruses in each cultivar, it was difficult to determine the major virus-causing disease symptoms. However, the proportion of viral reads in each library made it possible to detect the virus responsible for the disease symptoms in each cultivar. For example, while HPEV was the dominant virus in the P01 cultivar coinfected with HPEV and BBWV2, P01 (PR-9988 cultivar) did not display any disease symptoms. The viral abundance of BBWV2 was very low in P01. Recently, several pepper cultivars resistant to diseases such as phytophthora blight have been developed in Korea, and the disease-resistant cultivar is referred to as *Phytophthora* resistance (PR). In addition, we included several pepper cultivars that were resistant to anthracnose and viruses. For example, TS-Monster (P03) was developed for resistance against TSWV. Chegangtan (P06) is resistant to anthracnose. Caltan (P12) is resistant to CMV, TSWV, and anthracnose. Interestingly, most disease-resistant pepper cultivars showed no viral disease symptoms, despite being coinfected with several viruses.

Of the examined pepper cultivars, all four bell pepper cultivars displayed severe disease symptoms accompanied by improper growth and fruit production. To identify the major viruses causing disease symptoms in each bell pepper cultivar, we examined the dominant viruses based on the proportion of the viral reads. In P02, PCV2 was the most prominent virus, while CMV was the dominant virus in P09, P14, and P15. Based on this result, it was inferred that CMV may play a major role in the development of viral diseases in bell pepper plants. However, it was unclear whether PCV2 was the main virus causing disease symptoms in P02 because the PCV2-mediated disease symptoms have not been revealed to date. Moreover, BBWV2 and CMV may have coinfected the P02 cultivar. Although bell peppers are currently regarded as important horticultural plants in Korea, the viruses infecting bell peppers have not been intensively studied. In this study, we provided detailed information on viruses that infect major bell pepper cultivars.

BBWV2 is currently regarded as an important virus causing severe damage to pepper production in Korea. As expected, our results showed that BBWV2 was the dominant virus in 7 out of 15 cultivars: P03, P06, P07, P08, P10, P12, and P13. Therefore, it is important to prevent the spread of BBWV2 in pepper fields. Both CMV and BBWV2 can be transmitted by aphids and mechanically via sap. In addition, CMV is transmitted by seeds [20]; however, the seed transmission of BBWV2 has not been reported. Therefore, it may be of interest to examine the possible seed transmission of BBWV2 to prevent its spread in pepper plants.

Viral quasispecies is defined as population structure containing a large number of variant genomes caused by mutations [21]. In our pepper virome study, we revealed the viral quasispecies for BBWV2 and CMV based on the assembled viral genomes. To date, studies associated with viral quasispecies have been carried out by analyzing partial sequences of the target virus [22] or via RT-PCR followed by Sanger sequencing [23]. In contrast, we successfully identified viral quasispecies at the genome level through RNA sequencing. We showed that the viral quasispecies BBWV2 and CMV consisted of two and three RNA segments, respectively, according to their viral genome segment. We obtained the complete sequences for 24 BBWV2 RNA1 and 23 BBWV2 RNA2. The number of BBWV2 variants in each plant was similar, except for the P05 sample, which had two BBWV2 RNA1 variants and six BBWV2 RNA2 variants. For CMV, we obtained complete sequences for 15 RNA1, 13 RNA2, and 21 RNA3 variants. The number of CMV RNA variants in each pepper sample was different. For instance, none of the RNA1 and RNA2 variants were obtained from the P02 and P04 samples; however, five RNA3 variants were obtained from both samples. Phylogenetic analyses using the assembled BBWV2 and CMV variants revealed possible genetic relationships among the identified variants. In general, variants from the same sample were grouped, and some variants derived from different samples were closely related. For instance, the CMV RNA1 variants P04, P05-1, P11, P12, and P05-2 in group A were related. Similarly, at least 11 BBWV2 RNA2 variants in group A were closely related. These results suggest that the variants in the same group may have a common origin. In the case of CMV, all the variants in this study belonged to subgroup IA. The CMV isolates in subgroup I are tolerant to high temperatures and are frequently identified in regions with warmer climates [24]. Moreover, their viral disease symptoms are more severe than those in subgroup II [24]. The summer season in Korea is very hot and humid, and the disease symptoms caused by CMV in pepper plants were very severe, consistent with the results of a previous study [7].

In addition, other assembled virus genomes such as BWYV, BPEV, PCV1, and PCV2 obtained in this study were successfully employed for phylogenetic analyses to reveal genetic relationships with other known viral genomes.

The pepper plants used in this study originated from five different nurseries. Therefore, it is possible to track the origins of the identified viruses. However, we did not find any correlation between viral infections and the selected nurseries. Rather, most pepper cultivars displayed a cultivar-specific virome.

## 4. Materials and Methods

### 4.1. Plant Materials and Viral Disease Symptoms

Seedlings of 15 pepper cultivars were obtained from 5 nurseries (Table 1). The seedlings were grown in an open field. Leaves of a single pepper plant from each cultivar were harvested 1 month after transplantation and viral disease symptoms were observed. Collected pepper leaves were immediately frozen in liquid nitrogen.

### 4.2. Total RNA Extraction and Library Preparation

Frozen pepper leaf samples were ground using a mortar and pestle in liquid nitrogen. The ground powder (100 mg) was subjected to total RNA extraction using an RNeasy Plant Mini Kit (Qiagen, Hilden, Germany), according to the manufacturer’s instructions. The quality of the extracted total RNAs was measured using a Technologies 2100 Bioanalyzer (Agilent, Santa Clara, CA, USA). The total RNAs with RNA Integrity Number (RIN) values greater than or equal to 7 were used for library preparation. The libraries for RNA sequencing were prepared by depleting ribosomal RNAs from the total RNAs using TruSeq Stranded Total RNA with a Ribo-Zero Plant kit (Illumina, San Diego, CA, USA), according to the manufacturer’s instructions. A total of 15 libraries representing 15 pepper cultivars were prepared and, referred to as P01–P15.

### 4.3. RNA Sequencing and De Novo Transcriptome Assembly

The indexed libraries were paired-end-sequenced (150 bp × 2) using the Illumina HiSeq X system (Illumina). The raw RNA sequencing data were deposited in the sequence read archive (SRA) database in the National Center for Biotechnology Information (NCBI) with the respective accession numbers (Table S2). The raw data obtained as FASTQ files for each library were subjected to *de novo* transcriptome assembly using the Trinity assembler (version 2.13.2) with default parameters [25].

### 4.4. Identification of Viral Contigs and Calculation of Viral Reads

The assembled contigs from each library were subjected to a BLASTX search against the complete viral reference protein database derived from the NCBI using the DIAMOND program with an E-value of 1E-5 as cutoff [26]. BLASTX results in the DAA format were imported into the MEGAN6 program [27] to exclude the sequences derived from organisms other than viruses. Virus-associated contigs were extracted and subjected to a BLASTX search against NCBI’s non-redundant protein database using the same conditions. The BLASTX results in the DAA format were imported into the MEGAN6 program to filter out the non-viral sequences. Based on the BLASTX results and taxonomy classification using MEGAN6, the viral contigs were classified according to the virus species. The sequences of the viruses composed of multiple segments were classified according to the viral segments.

### 4.5. Assembly of Viral Genome and Virus Genome Annotation

Viral sequences covering all ORFs for each virus species were selected. The sequences were subjected to the NCBI ORFfinder [28]. Individual ORF in each viral genome was annotated according to a known reference viral genome. The assembled viral genome was named after the library. Several viral genomes from the same library were identified for certain viruses. All the assembled viral genomes were deposited in the NCBI GenBank database with their respective accession numbers (Table S3).

### 4.6. Construction of Phylogenetic Trees

The assembled viral genomes for each virus species were subjected to BLASTN against the NCBI nucleotide database. The known viral genome sequences for each of the identified virus species were extracted. The viral genome sequences from this study and the known viral genome sequences from GenBank were aligned using MAFFT version 7 with the auto option [29]. The aligned sequences were subjected to TrimAl for automated sequence trimming using the option automated1 [30]. The trimmed sequences were imported into the MEGA7 program to construct a phylogenetic tree [31]. A phylogenetic tree for each virus species was generated using maximum likelihood with 1000 bootstrap replicates. In the case of BWYV, BPEV, PCV1, and PCV2, the complete genome sequences from this study, as well as the corresponding known viral genome sequences, were used for phylogenetic tree construction. In contrast, for the phylogenetic tree construction of CMV and BBWV2, each viral segment from this study was used.

### 4.7. Designing of Primers to Detect Viruses Infecting Pepper Plants via RT-PCR

Primers were designed for recombinase amplification assays using the PrimedRPA program, to detect a total of nine viruses, namely CMV, BBWV2, BWYV, BPEV, HPEV, PCV1, PMMoV, PepMoV, and TSWV [32]. RT-PCR was performed, and the primer pairs that specifically amplified the target virus species were selected. The designed primers had amplicon sizes less than 200 bp for all the PCR products. The selected primer pairs could be also used for three different methods: RT-PCR, real-time RT-PCR, and RPA. However, in this paper, we only present the RT-PCR results.

### 4.8. RT-PCR and Agarose Gel Electrophoresis

RT-PCR was performed using a SoGent DiaStar One-Step RT-PCR Kit (Solgent, Daejeon, Korea). The PCR reaction contained the following elements: 1 µL of template RNA (100 ng), 1 µL of the forward primer (10 pmol/µL), 1 µL of the reverse primer (10 pmol/µL), 6 µL of 5× Band Doctor, 6 µL of 5× buffer and 2 µL of the enzyme mix adjusted to a final volume of 30 µL using RNase-free water. RT-PCR was carried out using the following conditions: cDNA synthesis at 50 °C for 30 min, initial denaturation at 95 °C for 15 min, denaturation at 95 °C for 20 s, annealing at 60 °C for 40 s, and extension at 72 °C for 40 s. The denaturation-to-extension steps were repeated 34 times, with a total of 35 cycles continued to a final extension at 72 °C for 5 min. The samples were maintained at 4 °C. The PCR products were separated via gel electrophoresis on 1.5% agarose gel in the presence of a TBE buffer. The PCR products were stained using ethidium bromide and visualized under ultraviolet light.

## 5. Conclusions

In this study, we carried out a comprehensive virome study of 15 pepper cultivars derived from 5 nurseries based in Korea. All four bell pepper cultivars showed severe viral disease symptoms; however, most hot pepper cultivars were either asymptomatic or had mild viral disease symptoms. RNA sequencing followed by bioinformatic analyses identified 1325 virus-associated contigs that were derived from 8 different RNA viruses. The proportion of viral reads in most pepper cultivars was relatively high, ranging from 0.04% to 24.5%. We found that BBWV2 and CMV were the most frequently identified viruses, followed by PVY. BBWV2 was the dominant virus in seven cultivars, whereas CMV was the dominant virus in five cultivars. Moreover, we reported, for the first time, the infection of BPEV in four different bell pepper cultivars in Korea. Although the pepper plants in this study originated from different nurseries, most pepper cultivars displayed a cultivar-specific virome. A total of 111 complete viral sequences for the 7 viruses were obtained. Using the obtained viral genome sequences, we revealed the genetic relationship between the identified viral isolates and known isolates for each virus. Moreover, we revealed quasispecies of BBWV2 and CMV in several pepper cultivars. We developed RT-PCR primers specific for nine RNA viruses infecting pepper plants, confirming the RNA sequencing results. Taken together, this study revealed the viromes of 15 pepper cultivars through RNA sequencing.

## Figures and Tables

**Table 1:** Summary of pepper cultivars used for virome study. A total of 15 different pepper cultivars derived from 5 nurseries indicated by region, were used for the virome study. Pepper cultivars were divided into three categories: red hot pepper, sweet pepper, and sweet paprika.

Index	Name	Category	Spicy	Region
P01	PR-9988	Red hot pepper	Hot	Hampyeong
P02	Mini paprika	Sweet paprika	Sweet	Ilsan
P03	TS-Monster	Red hot pepper	Hot	Hampyeong
P04	Boradori	Sweet pepper	Sweet	Ilsan
P05	Miin	Sweet pepper	Sweet	Ilsan
P06	Chegangtan	Red hot pepper	Hot	Pusan
P07	Dangjo	Sweet pepper	Sweet	Ilsan
P08	Ground cherry (Wrinkly)	Red hot pepper	Hot	Uljin
P09	Yellow paprika	Sweet paprika	Sweet	Anyang
P10	Vitamin	Sweet pepper	Sweet	Hampyeong
P11	Oi (Cucumber)	Sweet pepper	Sweet	Hampyeong
P12	Caltan	Red hot pepper	Hot	Anyang
P13	Cheongyang	Red hot pepper	Hot	Anyang
P14	Red paprika	Sweet paprika	Sweet	Anyang
P15	Green paprika	Sweet paprika	Sweet	Hampyeong

**Table 2:** Primer pairs used to detect viruses infecting pepper plants.

Viral Reference and Accession No.	Name	Position	Sequences	Size
18S rRNA of pepper	Ca18SrRNA-F1		CCGGTCCGCCTATGGTGTGCACCGGTCGTC	285
EF564281	Ca18SrRNA-R1		GCAGTTGTTCGTCTTTCATAAATCCAAGAA	
Cucumber mosaic virus RNA3	CMVs3-1507F1	1507–1536	TATTATGGTAAAAGGTTGTTGCTACCTGAT	155
D28780.1	CMVs3-1661R1	1661–1632	ACGGATAAGTCCGAGGAGGCAGAAACTTTA	
Pepper mild mottle virus	PMMoV-5881F1	5881–5910	CTGGTTTCAAAGTTTTCCGATATAATGCCG	179
MN496154.1	PMMoV-6059R1	6059–6030	ACTGGCCCTAATGGCCACCGTCGCATCATC	
Broad bean wilt virus 2 RNA2	BBWVs2-2463F1	2463–2492	TGCAAATTCTTGACTCGCTGCCCCATATCG	151
JX183230.1	BBWVs2-2613R1	2613–2584	GAAACATCATCCCCTCTTGCCGAGTCCAAA	
Tomato spotted wilt virus S segment	TSWV-S-2004F1	2004-2033	TAAGCAAGTTCTGCGAGTTTTGCCTGTTTT	150
KU179577.1	TSWV-S-2153R1	2153–5124	GAAGAAGGGGAAAGAGTATGCTGCTATACT	
Beet western yellows virus	BWYV-3892F1	3892–3921	CAGAACTCCGGCTCCATCGCTTACGAGCTG	169
MK307779.1	BWYV-4060R1	4060–4031	GGATCCTGAATTGGTCCTCGGCGACGTCGT	
Pepper mottle virus	PepMoV-8916F1	8916–8945	ATGGTTTGGTGCATTGAAAATGGCACGTCC	174
EU586122.1	PepMoV-9089R1	9089–9060	CATTTCTATATATGCCTCAGCCACATCAGA	
Hot pepper endornavirus	HPEV-13489F1	13489–13518	CTTTGATGCAAGTAAAGCAGACATATTGGC	183
NC_027920.1	HPEV-13671R1	13671–13642	AGGTGAACATTTAATCTGTTAATAGCATGC	
Bell pepper endornavirus	BPEV-13478F1	13478–13507	GTGGCAACTTATTTTGATGCAGACAAAGCA	188
NC_039216.1	BPEV-13665R1	13665–13636	ACATTTAGCCTGTTAATGGCATGTAGTTGC	
Pepper cryptic virus 1 RNA2	PCV1s2-1081F1	1081–1110	CCGCAACAGGTACAACACAACGAAGGAAGA	163
NC_037096.1	PCV1s2-1243R1	1243–1214	TTAGTCCTGATGACTGGAGGGAAGGTAACT	

## Data Availability

The raw data were deposited in the NCBI SRA database with the following accession numbers: SRR20337003–SRR20337017. The 111 viral genome sequences in this study were deposited in GenBank with the following accession numbers: ON759421–ON759531.

## Supplementary Materials

The supporting information can be downloaded at: https://www.mdpi.com/article/10.3390/ijms231810507/s1.
